# Leveraging Longitudinal Data to Sustain the Impact of Organ Donation Training: A Data Science Approach to Quality Monitoring

**DOI:** 10.1155/joot/8851596

**Published:** 2026-05-22

**Authors:** Omid Ghobadi, Nasrin Taherkhani, Roghaye Khasha, Ehsan Radi, Mahdi Shadnoush, Katayoun Najafizadeh

**Affiliations:** ^1^ Iranian Research Center of Organ Donation, Shahid Beheshti University of Medical Sciences, Tehran, Iran, sbmu.ac.ir; ^2^ Department of Information Technology Engineering, Payame Noor University (PNU), Tehran, Iran, pnu.ac.ir; ^3^ Department of Industrial Engineering & Management Systems, Amirkabir University of Technology, Tehran, Iran, aut.ac.ir; ^4^ Department of Clinical Nutrition & Food Technology, Shahid Beheshti University of Medical Sciences, Tehran, Iran, sbmu.ac.ir; ^5^ School of Medicine, Shahid Beheshti University of Medical Sciences, Tehran, Iran, sbmu.ac.ir

**Keywords:** data science, donation team training, IrOQS, IrOSS, longitudinal data, organ donation, quality control

## Abstract

**Background:**

The effectiveness of training organ donation teams in improving organ donation rates has been well established. However, studies have shown that this impact is often temporary and diminishes without ongoing support and quality control programs. This study aimed to evaluate the effects of initial training and the role of a structured quality control program in sustaining these improvements in Iran’s organ procurement units (OPUs).

**Method:**

A comprehensive training program, the Iranian OPUs Supporting System (IrOSS), was developed and implemented with the participation of 25 OPUs. Their organ donation (PMP) rates were recorded at baseline and monitored every 3 months for 6 months posttraining. Later, a quality control initiative, the Iranian OPUs Quality Control System (IrOQS), was introduced in 15 of these units. Through periodic assessments, weaknesses were identified and addressed via targeted interventions such as strategy improvements and retraining. The remaining 10 units served as a control group. PMP was reevaluated 3 and 6 months after the initial IrOQS implementation, with a final assessment 3 months after the second round. Longitudinal data were analyzed using advanced statistical techniques, including repeated‐measures ANOVA, difference‐in‐differences (DiD), linear mixed‐effects models (LMMs), trajectory analysis, and change point detection.

**Results:**

The mean PMP across all 25 OPUs increased from 5.31 before training to 8.05 six months after training. In the intervention group, the mean PMP increased to 13.06 after the first IrOQS phase, decreased to 9.84 over the next 6 months, and increased again to 15.6 after the second IrOQS phase. In contrast, while the control group showed an initial increase after training, their PMP gradually decreased over time. Statistical analyses showed that the initial training had a significant positive effect on performance, but these improvements were not sustained without repeated interventions. Path analyses also showed that units with lower initial performance responded most positively to training but tended to regress if retrained.

**Conclusion:**

The findings of this study suggest that initial training programs can lead to significant improvements in PMP. However, to maintain these gains, regular implementation of ongoing training programs is essential. Data science approaches and longitudinal data analysis can play a critical role in identifying performance patterns in the quality management of OPUs.

## 1. Introduction

In case of organ failure, transplantation is one of the most effective ways to save patients’ lives [1]. Worldwide, organ donation rates do not keep pace with the growing demand for transplants, leading to a continuously growing waiting list [2]. In Iran, approximately 7–10 people die each day because of a lack of organs for transplantation; currently, over 25,000 patients are on the waiting list for an organ transplant [3]. In 2020, Iran’s donor organ donation rate (PMP) was 7.8, which is low compared to the United States’ 38.03 and Spain’s 37.97 [4].

To increase the PMP and reduce the gap between the waiting list and the donation rate, it is essential to boost the organ donation rate. One of the most effective strategies to improve the donation rate from brain death is to enhance the success of organ donation teams and coordinators in obtaining consent from families. The existence of skilled and trained coordinators, who can follow patients from identification to the transplantation process, plays a crucial role in improving organ donation outcomes and saving lives [5].

Numerous studies indicate that providing training courses for healthcare professionals and organ donation coordinators significantly increases their success in requesting consent and, consequently, raises the organ donation rate [6–8]. For example, a study by Yao‐Mei Chuang et al. in 2021 found that education level, experience with organ donation, experience with transplant recipients, and participation in organ donation courses positively correlate with successful solicitation. The study suggested that mandatory training programs on requesting organ donation could improve the number of successful requests made by medical staff and coordinators [5]. Another study by Su Jin Heo et al. in 2021 in Korea reported the Donation Improvement Program (DIP), a program that educates and evaluates healthcare professionals about each stage of the donation process, promoting brain death recognition, increasing organ donation rates, and positively impacting healthcare professionals’ attitudes toward organ donation [9]. Similarly, a study focused on the training of organ procurement coordinators demonstrated that targeted communication strategies substantially improved consent rates, with a notable increase from 46.3% to 55.5% following an interactive training workshop. This suggests that enhancing coordinators’ communication skills can be an effective approach to increase consent rates, yet the need for further validation of the intervention’s long‐term efficacy remains apparent [10]. Healthcare professionals and organ donation coordinators play a key role in the donation and transplantation process [11].

Although training of coordinators has proven beneficial, other studies emphasize the necessity of maintaining training effectiveness over time. The evidence indicates that the impact of training interventions tends to wane unless ongoing support and quality improvement measures are integrated into the training framework. For example, the ETPOD initiative demonstrated significant improvements in organ donation parameters, reinforcing the idea that continuous evaluation and adjustment are critical to sustaining high organ donation rates [12]. Moreover, the importance of creating a robust quality control program has been underscored in various studies. Research indicates that comprehensive training, when paired with quality control initiatives, can lead to lasting improvements in donation metrics. The ONT’s experience exemplifies this, as their integrated approach resulted not only in increased referral rates and family consent but also in a reduction of losses due to cardiac arrest [13]. Such findings indicate that without a systematic approach to reevaluation and retraining, the benefits gained from initial training efforts may diminish over time, a challenge also faced by the organ procurement units (OPUs) in Iran.

In contrast to the focus on communication and quality control, some studies have pointed to the broader systemic factors affecting organ donation rates. For instance, the role of interprofessional collaborations and community awareness initiatives has been highlighted as essential for driving improvements in donation rates [14]. Although these approaches provide valuable insights into the multifaceted nature of organ donation, they often lack the specific actionable strategies that targeted training programs can offer.

In summary, although previous research has demonstrated the positive impact of educational programs on improving organ donation rates, most studies have focused primarily on short‐term outcomes and have not adequately addressed the sustainability of the effects of education. The present study not only evaluates the immediate effects of an initial standardized educational program (Iranian OPUs Quality Control System [IrOSS]) but also introduces and evaluates a secondary quality control system (Iranian OPUs Quality Control System [IrOQS]) that was implemented to maintain and improve the performance of educational programs over time. By utilizing longitudinal data analysis and advanced statistical techniques, this study provides a data‐driven framework for continuous performance improvement in OPUs.

## 2. Method

In this study, a comprehensive training program called IrOSS was developed to evaluate the impact of training on the performance of OPUs. The aim was to enhance the efficiency of these units and improve both the quantity and quality of organ donation in Iran.

The educational topics of this program included modern techniques for identifying suspected brain death cases, diagnosis of brain death, organizational and legal coordination, maintenance protocol for brain‐dead patients, necessary measures in the ICU, criteria for selecting appropriate cases and organs for transplantation, principles for interacting with families of brain‐dead patients, and techniques for obtaining family consent for organ donation.

To maximize the effectiveness of the training, diverse teaching methods were employed, including lectures with related topics and sharing of experiences by instructors, group activities, practical workshops, think tank discussions, game‐based learning, simulations, and Q&A panels. In addition, members of the brain death confirmation team—including anesthesiologists, neurosurgeons, neurologists, emergency medicine specialists, and forensic medicine experts—were invited to participate in the training and receive the latest guidelines on brain death confirmation and organ procurement process. Participant understanding was evaluated through a daily test at the end of each day, covering the topics presented that day. A comprehensive final assessment was also conducted on the last day, encompassing all course subjects.

In Iran, each university of medical sciences is mandated to operate its own organ donation system within one of its affiliated hospitals, preferably the trauma center of that university. Management of organ donation activities in other city hospitals falls under the jurisdiction of this OPU. Currently, 56 OPUs are active across the country, overseeing organ donation processes in various hospitals. For this study, 25 counties with low organ donation rates, each covered by an existing OPU, were selected to participate in the training program. Hospitals within each OPU’s jurisdiction were evaluated based on their facilities and capacity, and personnel from these hospitals were selected for participation according to the criteria outlined in Table 1.

**TABLE 1:** Treatment staff participating in the IrOSS course according to the type of hospital.

Type of hospital personnel	Hospitals with neurosurgery, neurology, and ICU	Hospitals with neurology and ICU	Hospitals with ICU and without neurosurgery and neurology	Hospitals without neurosurgery, neurology, and ICU
Matron	✔	✔	✔	✔
Head of nurses of ICUs and ERs	✔	✔	✔	
Educational supervisor	✔	✔	✔	
Emergency nurses	✔	✔	✔	✔
Surgical ICU nurses	✔			
Internal ICU nurses	✔	✔	✔	
Emergency room nurses	✔	✔	✔	✔
Operating room technicians	✔	✔	✔	✔
Anesthesiology technicians	✔	✔	✔	✔
Emergency members	✔	✔	✔	✔
Psychologists and social workers	✔	✔	✔	✔

Abbreviations: ER = emergency room, ICU = intensive care unit.

**FIGURE 1:**
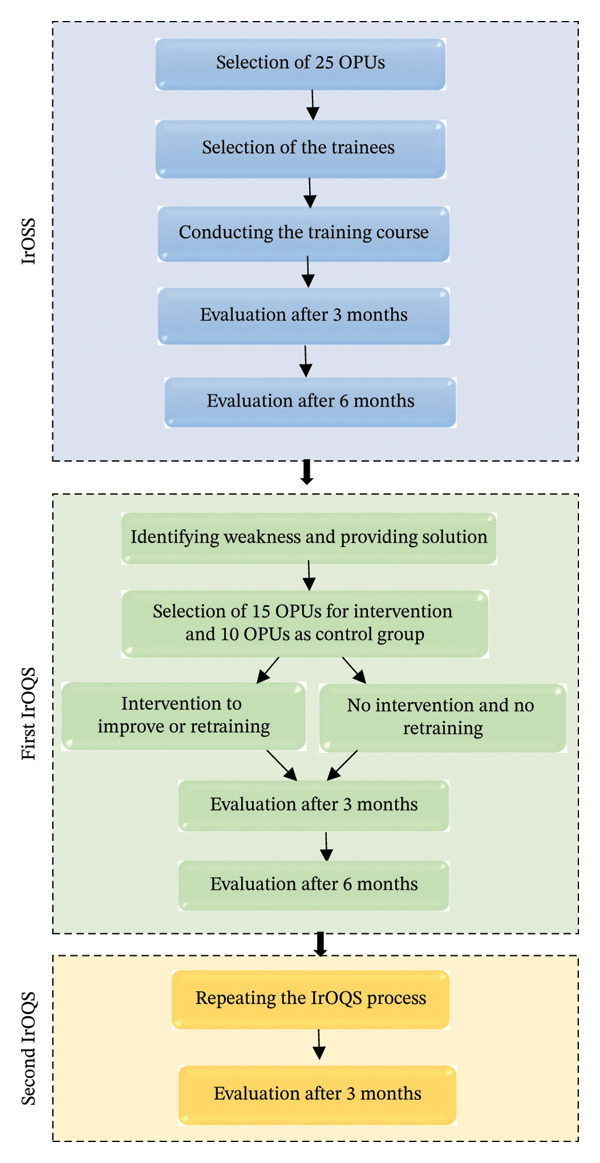
The stages of implementation and evaluation of IrOSS and IrOQS.

During the first 6 months after the training, monitoring occurred at 3‐month intervals, and the PMP of each OPU was recorded. Six months after the implementation of IrOSS and the conducted assessments, a program called IrOQS was developed and implemented in 15 of these OPUs. According to the monitoring, the weaknesses of these 15 OPUs were investigated, solutions were provided to improve the conditions, or retraining courses were held. The remaining 10 OPUs served as the control group. Following the implementation of IrOQS, the PMP of all participating OPUs was reevaluated at 3 and 6 months. To ensure an accurate assessment of the program’s impact, IrOQS was reapplied, and the PMP was again evaluated 3 months later. The stages of implementing and assessing both the IrOSS and IrOQS projects are illustrated in Figure 1. Notably, this study was approved by the Ethics Committee of Shahid Beheshti University of Medical Sciences (Approval Code: IR.SBMU.RETECH.REC.1402.824).

In this study, advanced data mining and statistical methods were employed to analyze the longitudinal performance data of OPUs. These methods were selected based on the repeated‐measures nature of the data and the study’s objectives, which included assessing long‐term changes, identifying performance patterns, and comparing intervention and control groups.

### 2.1. Difference‐in‐Differences (DiD) Analysis

To evaluate the true effect of the IrOQS program, the DiD approach has been utilized. This method helps isolate the actual impact of the intervention from temporal and environmental confounders. Our goal is to determine whether the observed increase in PMP in the intervention group after IrOQS is truly attributable to the intervention itself or merely the result of time or other external factors. DiD is particularly effective in quasiexperimental studies with limited sample sizes, allowing for better control of external variables. To achieve this goal and better understand the changes in PMP within the intervention and control groups, we separated the data before and after the IrOQS intervention and created an interaction variable. By creating this interaction variable between group (intervention/control) and time (before/after IrOQS), it is possible to identify changes attributable specifically to the intervention. The interaction term in a DiD analysis is a powerful statistical tool that helps us identify the true effect of the intervention relative to time and the control group. The model includes three key variables: Group (intervention vs. control), Time (pre‐ vs. post‐IrOQS), and their interaction term (Interaction = Group × Post_Treatment). The variables were coded as follows:

• Post_Treatment = 0: before the implementation of IrOQS
• Post_Treatment = 1: after the implementation of IrOQS

The interaction variable takes the value 1 only when the observation belongs to the intervention group and is after the IrOQS implementation. In other words, this variable equals 1 only when both the intervention is present and the time is postintervention. This approach is more robust than a simple pre‐ or postcomparison because the groups may have been different from the start, or all OPUs might improve over time due to natural growth. Additionally, the control group may experience improvement or decline due to other external factors. Therefore, this analysis helps isolate the true effect of the intervention. After constructing the new dataset with the interaction variable, the regression line is fitted to the data using the dependent variable PMP. The regression equation was formulated as PMP = β_0_ + β_1_·Group + β_2_·Post_Treatment + β_3_·(Group × Post_Treatment) + *ε*, where the coefficient β_3_ represents the DiD estimator capturing the differential change in PMP attributable specifically to the IrOQS intervention after controlling for preexisting group differences and broader time‐related changes affecting all OPUs.

### 2.2. Linear Mixed‐Effects Models (LMMs)

In the next stage of our analysis, we aim to investigate factors influencing improvements in PMP scores while accounting for the data’s dependency structure (i.e., repeated measurements for each OPU over time). To this end, we utilize an LMM. Given that our data are longitudinal, each OPU is measured at multiple time points and the observations within each unit are not independent. While simple linear models (OLS) assume independence of observations, this assumption does not hold in our context. Therefore, the LMM is a more appropriate approach for our analysis. This model allows us to account for within‐unit variation and ultimately assess the impact of IrOSS and IrOQS interventions while controlling for baseline performance and group assignment.

To implement this analysis, a new variable named Pre_Training_PMP was derived from the PMP data prior to the implementation of the IrOSS program for each OPU. This variable represents the organ donation rates (PMP) before the start of the intervention and serves as a baseline indicator of each unit’s initial performance. It remains constant across all subsequent time points, reflecting the preintervention status of each OPU. Pre_Training_PMP not only serves as a strong predictor of future performance but also acts as a control variable in the LMM. Including this variable in the regression model allows us to adjust for baseline differences across OPUs and thus to more accurately estimate the true effects of the IrOSS and IrOQS interventions.

The final LMM incorporated three fixed effects: (1) Time_Point, a categorical variable with five postbaseline measurement occasions (3 months after IrOSS, 6 months after IrOSS/before IrOQS, 3 months after the first IrOQS implementation, 6 months after the first IrOQS implementation, and 3 months after the second IrOQS implementation), with the baseline measurement serving as the reference category; (2) Pre_Training_PMP, a continuous covariate representing each OPU’s organ donation rate before any intervention; and (3) Group, a binary indicator distinguishing the intervention group from the control group. To address the nonindependence of repeated measurements within each OPU, a random intercept was included for every unit, assuming a normal distribution with mean zero and variance *σ* ^2^b.

### 2.3. Trajectory Analysis

To uncover trends and patterns in OPU performance over time, trajectory analysis has been conducted. For this purpose, OPUs were stratified into three performance clusters based on pre‐IrOSS PMP values using the following a priori thresholds: (1) high performers (PMP ≥ 10), (2) medium performers (5 ≤ PMP < 10), and (3) low performers (PMP < 5). Mean PMP trajectories were then computed for each cluster across six measurement occasions: baseline (before IrOSS), 3 months after IrOSS, 6 months after IrOSS (before IrOQS), 3 months after first IrOQS, 6 months after first IrOQS, and 3 months after second IrOQS. This approach helps determine whether baseline performance levels influenced responsiveness to training and quality interventions.

### 2.4. Change Point Detection

Change point detection methods have been used to identify the exact time points at which significant changes in OPU performance occurred due to the interventions. This technique does not rely on prior assumptions and detects time points where statistically significant shifts in PMP took place. This method allows the identification of change milestones and the impact of IrOSS and IrOQS interventions on each OPU.

In our study, for each OPU, a time series of PMP values is available. The goal is to identify change points, i.e., where the data pattern changes. Two criteria are used to identify these points:

⁃ Rolling mean: Using a sliding window of size 3, the mean of the last three values is calculated at each point. This helps major shifts in the data trend to be identified.
⁃ Rolling standard deviation: This metric indicates how much the data varies around the mean. A sudden increase in the standard deviation signals abnormal changes, which are identified as change points where OPU performance has changed. If the moving standard deviation is greater than the threshold equal to the average of deviations, the point is recognized as a change point. This is a simple yet effective method for detecting sudden changes in time‐series data.

Also, for an improvement to be considered sustainable, two conditions must be met:

⁃ There must be at least two change points, indicating that the improvement was not a one‐time event but occurred multiple times.
⁃ The latest change must have occurred in the final period, suggesting that the improvement was maintained until the end of the measurement period.

From a methodological perspective, we integrated data science tools and statistical modeling to assess the long‐term effects of training in OPUs. Unlike previous studies that focused primarily on short‐term outcomes, this study uses longitudinal data to provide a data‐driven framework for ongoing monitoring to achieve sustainable improvement. This approach can be applied to other education and healthcare systems.

## 3. Results

In this section, the results of IrOSS and IrOQS are presented separately. Table 2 displays the PMP of 25 selected OPUs at three time points: before IrOSS, 3 months after IrOSS, and 6 months after IrOSS.

**TABLE 2:** PMP of OPUs at 3 time points before IrOSS, 3 months after IrOSS, and 6 months after IrOSS.

ID	OPUs	PMP (before IrOSS)	PMP (3 months after IrOSS)	PMP (6 months after IrOSS)
1	Bandar Abbess	0.5	13.3	6.6
2	Ardebil	15.45	25.4	21.8
3	Kermanshah	2.5	10.5	4.2
4	Rasht	10.4	16	8
5	Khorramabad	3	17.7	6.6
6	Ahvaz	2.6	9	4.3
7	Bushehr	1	11	7.2
8	Qazvin	8.3	20	13.4
9	Qom	2.3	11	8.7
10	Shahrekord	16	28	21
11	Kashan	2.4	8.9	6.1
12	Esfahan	6.3	8.5	6.2
13	Hamedan	6.3	8	5.8
14	Sanandaj	0.62	2.6	1.8
15	Sari	6.7	10	7.8
16	Tabriz	3.6	6	6.8
17	Urmia	4	4.4	3.3
18	Gorgan	0	0	2.9
19	Zahedan	2.3	2.3	0
20	Zabol	0	0	0
21	Rafsanjan	0	5.6	11
22	Sirjan	0	23	11.4
23	Jiroft	0	1.3	0
24	Birjand	7.5	10	5
25	Yasouj	31	40	31.4

As observed, most OPUs showed an increase in PMP 3 months after IrOSS. However, by 6 months after the training, a decline in PMP was noted across most units. Notably, Tabriz and Refsanjan maintained an upward trend throughout. These findings suggest that the training alone may not ensure a sustained long‐term improvement in performance.

In the next phase of the study, after 6 months, the reasons for the decline in PMP were investigated, as described in the research methodology. Subsequently, the IrOQS was implemented. Fifteen OPUs were designated as the intervention group, while ten OPUs served as the control group. Interventions included performance improvements and retraining; no interventions were conducted in the control group. Evaluations were repeated at 3 and 6 months following IrOQS implementation. For the final assessment, IrOQS was performed again after 6 months from the first round, with procedures identical to the initial assessment; this time, only a 3‐month follow‐up was conducted. The findings from these evaluations are summarized in Tables 3 and 4.

**TABLE 3:** PMP of OPUs after first and second IrOQS in the intervention group.

ID	OPUs	PMP (before IrOQS)	PMP (3 months after first IrOQS)	PMP (6 months after first IrOQS)	PMP (3 months after second IrOQS)
1	Bandar Abbess	6.6	13.3	11.1	17.7
2	Ardebil	21.8	27.2	19.5	28.6
3	Kermanshah	4.2	8	6.3	12.6
4	Rasht	8	11.2	7.2	14
5	Khorramabad	6.6	11.5	8	16
6	Ahvaz	4.3	10.2	7.4	12
7	Bushehr	7.2	12.4	9	14.3
8	Qazvin	13.4	16.2	11.5	18.7
9	Qom	8.7	13	9.3	12
10	Shahrekord	21	30	24.3	33.2
11	Kashan	6.1	9.6	8	16.3
12	Esfahan	6.2	10.7	9.3	13.3
13	Hamedan	5.8	11	8.1	8.8
14	Sanandaj	1.8	3.4	2.4	6.8
15	Sari	7.8	8.2	6.3	9.8

**TABLE 4:** PMP of OPUs after first and second IrOQS in the control group.

ID	OPUs	PMP (before IrOQS)	PMP (3 months after first IrOQS)	PMP (6 months after first IrOQS)	PMP (3 months after second IrOQS)
1	Tabriz	6.8	2.22	3	3
2	Urmia	3.3	3	3	5.3
3	Gorgan	2.9	0	1.6	1.6
4	Zahedan	0	0	0	0.8
5	Zabol	0	0	0	0
6	Rafsanjan	11	33.3	22	15
7	Sirjan	11.4	8	6.2	0
8	Jiroft	0	0	0	0
9	Birjand	5	12.5	8.33	6.26
10	Yasouj	31.4	27	26.7	11.4

As shown in Tables 3 and 4, interventions and retraining were associated with an increasing trend in PMP during the 3 months following the intervention. However, this upward trend reversed after 3 months. Conversely, in the control group—where no intervention took place—the PMP continued to decline.

**FIGURE 2:**
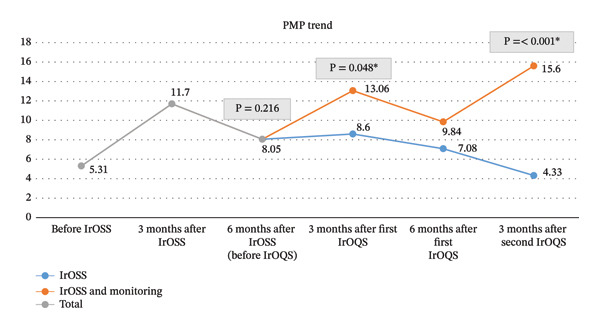
PMP average trend in the intervention and control groups.

Figure 2 illustrates the changes in the average PMP of both groups (intervention and control) over time, highlighting the impact of training and monitoring. The black line represents the average PMP of the 25 OPUs during the initial three periods, during which only training was administered. The increase from an average PMP of 5.31 (pretraining) to 8.05 (6 months after training) demonstrates the positive effect of training over time, despite some fluctuations. The trend was upward during the first 3 months and then declined in the subsequent 3 months.

The orange line depicts the PMP trend of the 15 OPUs in the intervention group. As shown, during all monitoring intervals, the PMP significantly increased—from 8.05 (before the first IrOQS) to 13.06 (3 months after the first IrOQS). The subsequent 3 months saw a decrease to 9.84, but then PMP increased again, reaching 15.6 at 3 months after the second IrOQS. These results highlight the importance of continuous monitoring at shorter intervals—such as quarterly—rather than 6‐month periods.

The blue line in Figure 2 illustrates the PMP trend of the control group. Although there was an upward trend from 5.31 (pretraining) to 7.08 (twelve months posttraining), this improvement was not sustained, and the PMP gradually declined to 4.33 by the end of the period. Overall, the PMP values of the control group remained consistently lower than those of the intervention group at comparable time points.

A repeated‐measures ANOVA was used to confirm the above results. This test is used to compare changes in PMP over time across all OPUs and to evaluate the statistical significance of the effects of IrOSS and IrOQS on PMP. The aim is to determine whether the changes before and after the interventions are statistically significant. Results indicated a statistically significant effect of time on PMP (*F*(5, 120) = 8.49, *p* < 0.001), confirming that changes in organ donation rates throughout the study were not random but systematically associated with IrOSS and IrOQS interventions. The average PMP increased from 5.31 before training to 11.7 at 3 months post‐IrOSS, followed by a decline to 8.05 at 6 months, suggesting that initial training led to temporary improvements. Among units receiving IrOQS, mean PMP further increased to 13.06 at 3 months after the first round, decreased slightly to 9.84 at 6 months, and then rose again to 15.6 following the second IrOQS implementation. In contrast, the control group showed an initial increase following IrOSS but failed to sustain gains, returning toward baseline levels over time. In summary, the ANOVA results confirm that training alone has only a temporary effect. The findings indicate that regular retraining and supervisory programs (such as IrOQS) can sustain and even enhance the effects of training. Moreover, a valuable finding is that there is a significant difference over time between the responses of the intervention and control groups.

### 3.1. DiD Analysis

A DiD regression was performed to isolate the causal effect of IrOQS from secular trends. A summary of the results of this analysis is presented in Table 5.

**TABLE 5:** Difference‐in‐Differences (DiD) analysis.

Variable	Coef	Std err	*t*	*p* > |*t*|	[0.025	0.975]
Interaction	2.6294	3.804	0.691	0.491	−4.931	10.19

The estimated coefficient (β) for the defined variable was 2.6294, indicating that, on average, the intervention group improved 2.63 units more than the control group in the post‐IrOQS period. However, the *p* value associated with this estimate is 0.491, indicating that the difference in the effect of IrOQS between the intervention and control groups is not statistically significant. In other words, although the mean improvement was higher in the intervention group, the magnitude of this difference was not large enough to be attributed with high statistical confidence to the effect of IrOQS. Several factors may have contributed to the lack of statistical significance:

− The available sample size was small: Only 15 intervention units and 10 control units were available.
− There is great variation in the data of some units, such as Yasouj and Rafsanjan.
− Contextual factors and cultural and regional differences in the units under study may have affected the effectiveness of the intervention.
− The IrOQS program may not have been implemented similarly in all units under study.

As a result, the DiD analysis shows that the IrOQS intervention did not significantly increase the effectiveness of IrOSS training compared to the control group. However, this does not mean that IrOQS was ineffective. Rather, it may be that the effect was not strong enough to be detectable by statistical models due to the limited sample size and heterogeneity of the data. To substantiate this claim, the results of the previous analysis are summarized in Table 6. Even though there was no statistical significance, the observed pattern suggests that the training effects improved in the intervention group, while the control group showed a downward trend. Therefore, while the descriptive data indicate improvement, the statistical evidence is insufficient to confirm this effect.

**TABLE 6:** The mean PMP score of OPUs after the implementation of IrOQS.

Group	Before IrOQS	3 months after first IrOQS	6 months after first IrOQS	3 months after second IrOQS
Intervention	8.05	13.06	9.84	15.6
Control	7.08	2.22	3	4.33

### 3.2. LMM

Table 7 provides a summary of the results from the LMM. The results of the LMM showed that all time points measured after the training (IrOSS, IrOQS) had a significant increase in PMP compared to before the training (before IrOSS). The greatest increases were observed at 3 months after IrOSS (*β* = 6.39, *p* ≤ 0.001) and 3 months after the second IrOQS (*β* = 5.79, *p* ≤ 0.001).

**TABLE 7:** Summary of the results from the linear mixed‐effects model.

Variable	Coef.	*z*‐value	*p* value	Interpretation
Time_Point: 3 mo after IrOSS	6.389	5.317	≤ 0.001	A highly significant increase in PMP was observed after the first IrOSS training session. This indicates that IrOSS had a strong and positive impact.
Time_Point: 6 mo after IrOSS (before IrOQS)	2.741	2.281	0.023	A moderately significant increase in PMP was observed 6 months after IrOSS. This increase remains statistically significant but has slowed down compared to the previous 3‐month period.
Time_Point: 3 mo after first IrOQS	5.966	4.965	≤ 0.001	A highly significant increase in PMP was observed after the first IrOQS course. This indicates that IrOQS has been as effective as IrOSS.
Time_Point: 6 mo after first IrOQS	3.430	2.855	0.004	A moderately significant increase in PMP was observed 6 months after the first IrOQS. This indicates that the effect of IrOQS persists to some extent.
Time_Point: 3 mo after second IrOQS	5.788	4.816	≤ 0.001	A highly significant increase in PMP was observed after the second IrOQS course. This indicates that repeating IrOQS continues to have a positive effect.
Pre_Training_PMP	0.840	7.295	≤ 0.001	Positive and significant effect of PMP before training on PMP after training. This indicates that OPUs with higher performance before training also performed better after training.
Group	3.473	2.152	0.031	A significant difference was observed between the groups. This indicates that the intervention group had a higher average PMP compared to the control group.

Moreover, pretraining performance (Pre_Training_PMP) had a positive and significant effect (*β* = 0.84, *p* ≤ 0.001) on subsequent performance, indicating that OPUs with higher initial performance also performed better after training. In addition, the intervention group performed better than the control group (*β* = 3.47, *p* = 0.031), suggesting the effectiveness of the intervention.

The difference between 3 months and 6 months may suggest that the training’s effect is initially strong but gradually diminishes over time. Repeating the training (IrOQS) can effectively compensate for this decline. The positive effect of Pre_Training_PMP suggests that a better selection of OPUs before the start of training might help improve outcomes.

### 3.3. Trajectory Analysis

In this section, we aim to address the research question: Is the baseline performance of OPUs (before IrOSS) associated with their responsiveness to the IrOSS and IrOQS interventions? To explore this, we analyzed data from 25 OPUs. For each unit, PMP was measured at multiple time points: Before IrOSS, 3 months after IrOSS, 6 months after IrOSS (or before IrOQS), 3 months after IrOQS, 6 months after IrOQS, and 3 months after the second IrOQS.

The data were restructured in a time‐series format, where each OPU has a longitudinal trajectory of PMP values across the above time points. Based on the baseline PMP (before IrOSS), OPUs were categorized into three groups: low, medium, and high initial performance. We then examined and visualized the trajectory of PMP changes over time for each group to assess whether baseline performance is associated with the pattern of improvement following the interventions. The classification of OPUs into three groups based on their pre‐IrOSS PMP values is presented in Table 8.

**TABLE 8:** Classification of OPUs into three groups based on pre‐IrOSS PMP values.

Cluster names	PMP range	OPUs
High performers (Cluster #1)	PMP ≥ 10	Shahrekord, Yasouj, Ardebil, Rasht
Medium performers (Cluster #2)	5 ≤ PMP < 10	Esfahan, Sari, Hamedan, Qazvin, Birjand
Low performers (Cluster #3)	PMP < 5	Bandar Abbas, Kermanshah, Kashan, Sanandaj, Ahvaz, Bushehr, Jiroft, Zahedan, Zabol, Gorgan, Khorramabad, Qom, Tabriz, Urmia, Rafsanjan, Sirjan

**FIGURE 3:**
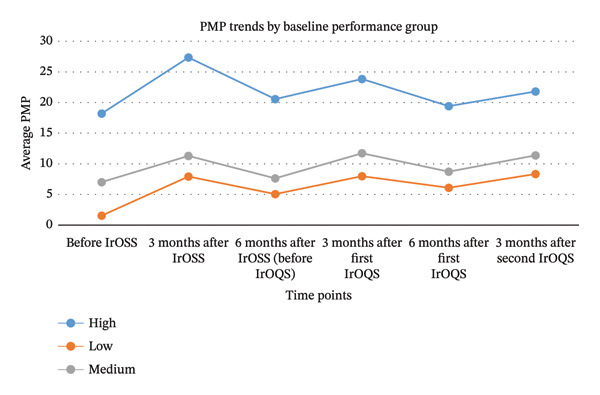
Trajectory analysis results for the three defined OPU clusters based on their initial performance.

Then, for each of the six defined time points, the average PMP of the OPUs within each group was calculated. The results are presented in Figure 3.

− In the first stage (before IrOSS), the average PMP in Cluster 1 was 18.21, in Cluster 2 was 7.02, and in Cluster 3 was 1.55.
− In the second stage (3 months after IrOSS), PMP increased in Cluster 1 (27.35) and Cluster 2 (11.3), while Cluster 3 experienced a remarkable improvement (7.91), with an increase of 408%. IrOSS was effective for all clusters, but the Low Performers showed the most significant growth. High Performers were already doing well, but still showed a positive shift.
− In the third stage (6 months after IrOSS), all clusters showed a decline in performance. This indicates that, without continued interventions, the initial improvements gained from training fade over time. One‐time training is not sufficient. Indeed, all clusters experienced a decline before the IrOQS intervention, even those with previously strong performance.
− In the fifth stage (3 months after the first IrOQS), Cluster 3, previously falling behind, showed a recovery with a PMP of 7.99. Cluster 2 slightly improved, and Cluster 1 had a small increase but still remained below its previous peak. Overall, IrOQS led to renewed improvements, especially among Low Performers.
− In the sixth stage (6 months after the first IrOQS), all three clusters showed a decline in mean PMP, suggesting that the effect of IrOQS was temporary and ongoing interventions are necessary.
− In the seventh stage (3 months after the second IrOQS), Cluster 1 showed a slight improvement, while Clusters 2 and 3 had more noticeable gains. This indicates that repeating the quality program helped reestablish the benefits of prior training.

The trajectory analysis indicates that the low performers cluster (e.g., Bandar Abbas, and Kermanshah) showed the best response to the IrOSS program but regressed again without the IrOQS intervention. The high performers cluster (e.g., Shahrekord and Yasouj) had a high initial performance but experienced a decline in the absence of IrOQS. The medium performers cluster exhibited a moderate response.

### 3.4. Change Point Detection

The results of the change point detection analysis are presented in Table 9. The “First Change Point” column indicates the earliest time point at which a significant performance improvement was detected, while the “Last Change Point” reflects the most recent time point where a change occurred. The “IrOSS Effect?” column specifies whether the observed changes occurred within the first two follow‐up periods (i.e., 3 months or 6 months after IrOSS), suggesting an initial response to the IrOSS intervention. Similarly, the “IrOQS Effect?” column indicates whether changes were detected during the periods following the IrOQS training (i.e., 3 months after first IrOQS, 6 months after first IrOQS, or 3 months after second IrOQS). Finally, the “Sustained?” column identifies whether the improvements were maintained over time, defined as having at least two change points and a final change occurring at the last observation period.

**TABLE 9:** Response patterns and change point detection across OPU performance groups.

OPU	Performance group	First change point	Last change point	IrOSS effect?	IrOQS effect?	Sustained?
Bandar Abbas	Low	3 months after IrOSS	3 months after IrOSS	Yes	No	No
Jiroft		3 months after IrOSS	6 months after IrOSS (before IrOQS)	Yes	No	No
Kermanshah		3 months after IrOSS	6 months after first IrOQS	Yes	Yes	No
Sirjan		3 months after IrOSS	6 months after IrOSS (before IrOQS)	Yes	No	No
Khorramabad		3 months after IrOSS	6 months after IrOSS (before IrOQS)	Yes	No	No
Ahvaz		3 months after IrOSS	3 months after first IrOQS	Yes	Yes	No
Bushehr		3 months after IrOSS	3 months after IrOSS	Yes	No	No
Rafsanjan		6 months after IrOSS (before IrOQS)	3 months after first IrOQS	Yes	Yes	No
Qom		3 months after IrOSS	3 months after IrOSS	Yes	No	No
Zabol		No	No	No	No	No
Kashan		3 months after IrOSS	6 months after first IrOQS	Yes	Yes	No
Zahedan		3 months after IrOSS	6 months after IrOSS (before IrOQS)	Yes	No	No
Gorgan		3 months after IrOSS	3 months after first IrOQS	Yes	Yes	No
Sanandaj		6 months after first IrOQS	6 months after first IrOQS	No	Yes	No
Urmia		6 months after IrOSS (before IrOQS)	6 months after first IrOQS	Yes	Yes	No
Tabriz		6 months after IrOSS (before IrOQS)	3 months after first IrOQS	Yes	Yes	No

Hamedan	Medium	6 months after IrOSS (before IrOQS)	3 months after first IrOQS	Yes	Yes	No
Birjand		6 months after IrOSS (before IrOQS)	3 months after first IrOQS	Yes	Yes	No
Esfahan		6 months after IrOSS (before IrOQS)	6 months after first IrOQS	Yes	Yes	No
Qazvin		3 months after IrOSS	3 months after IrOSS	Yes	Yes	No
Sari		3 months after IrOSS	6 months after first IrOQS	Yes	Yes	No

Shahrekord	High	3 months after IrOSS	3 months after IrOSS	Yes	No	No
Rasht		3 months after IrOSS	6 months after first IrOQS	Yes	Yes	No
Ardebil		3 months after IrOSS	6 months after first IrOQS	Yes	Yes	No
Yasouj		6 months after IrOSS (before IrOQS)	6 months after first IrOQS	Yes	Yes	No

The change point detection analysis was conducted on 25 OPUs to identify the sustainability of performance improvements following the IrOSS and IrOQS training programs. Results indicated that 92% of units demonstrated a significant response to IrOSS. In contrast, 64% of OPUs showed a subsequent response to IrOQS. Notably, none of the OPUs met the two defined criteria for sustained improvement. These findings suggest that while the IrOSS intervention produced initial improvements in most units, these effects were not sustained over time, and the impact of IrOQS was also relatively smaller.

The results of the analyses showed that all but two of the high‐performing OPUs (PMP ≥ 10 at baseline) responded to the IrOSS intervention. It is noteworthy that only one of these units did not show a subsequent change point after IrOQS training. None of the high‐performing OPUs met the criteria defined for sustained improvement. These findings suggest that although IrOSS can produce change in high‐performing units, ongoing strategies are needed to maintain these improvements over time.

Among medium‐performing OPUs (5 ≤ PMP < 10), all units responded to the IrOSS and IrOQS interventions, indicating their effectiveness. However, no unit displayed sustained improvements over time, indicating that the positive effects of training were not consistently preserved. This pattern emphasizes the value of IrOSS as an initial program and suggests that IrOQS has also been effective as a complementary intervention for this group, although implementation of reinforcement mechanisms to consolidate gains is essential to achieve sustainable improvement.

In low‐performing units (PMP < 5), 14 of 16 units (87.5%) showed initial improvement after IrOSS, indicating that first training can effectively bring about change even in low‐performing units. Eight units (50%) also responded to the IrOQS, indicating relatively moderate responsiveness to secondary training in this group. Importantly, none of the low‐performing units had sustained improvement. These results demonstrate the capacity of IrOSS to make short‐term gains among low‐performing units, but follow‐up strategies are needed to sustain these improvements in the long term.

## 4. Discussion

This study aimed to investigate the longitudinal effects of an initial training program (IrOSS) and a secondary quality control intervention (IrOQS) on the performance of OPUs. The results showed that the observed changes in donation rates were not random and the implemented programs showed a statistically significant temporal impact on improving the PMP rate (*F* (5, 120) = 8.49, *p* < 0.001). The results obtained in this study support the hypothesis that although initial training can significantly increase performance, its effects diminish over time in the absence of ongoing reinforcement programs such as IrOQS.

To evaluate the impact of IrOQS between intervention and control groups, a DiD analysis was conducted. The interaction term between the time variable (before and after IrOQS) and the group variable (intervention vs. control) was not statistically significant (*β* = 2.63, *p* = 0.491). This result indicates that the observed changes in units cannot be definitively attributed to the effect of the IrOQS intervention and may instead reflect secular trends, natural growth, or other external factors affecting organ donation rates over time. However, descriptive trends in PMP showed more sustained improvement in the intervention group, while the control group experienced a gradual decline. While these patterns are suggestive, the nonsignificant DiD estimate precludes causal claims about IrOQS effectiveness. This limitation likely reflects the modest sample size (15 intervention vs. 10 control units) and high interunit heterogeneity. With a larger sample size, more frequent implementation, or improved study design, a true intervention effect, if it exists, might reach statistical significance.

The LLM results showed that the IrOSS and IrOQS training programs had a significant impact on improving the performance of OPUs. All implemented training programs resulted in an increase in the PMP index. Among them, the greatest improvement was observed in the period 3 months after IrOSS and also 3 months after the second IrOQS session. These findings indicate that periodic training not only leads to immediate performance improvements but also maintains their effects when repeated. The pretraining performance index (Pre_Training_PMP) played an important role in determining posttraining performance levels. In other words, OPUs with higher initial performance experienced greater improvement after undergoing the training programs. This indicates that selecting OPUs based on their performance indicators before training begins plays an important role in improving training outcomes. Also, the comparison between the intervention and control groups showed that the intervention group performed better (*β* = 3.47, *p* = 0.031), indicating the effectiveness of the intervention. This finding emphasizes the need to implement similar approaches in other centers. While the increase in PMP was significant at 3 months after IrOSS and IrOQS, it decreased at 6‐month follow‐up. This pattern also demonstrates that without regular reinforcement and repetition, the effects of training may diminish over time. Therefore, implementing periodic training programs can play a key role in maintaining these improvements. Another key finding of this analysis was that repeated training (IrOQS) led to improved performance once again. This suggests that retraining can not only compensate for the gradual decline in the effects of initial training but also improve performance beyond previous levels.

A trajectory analysis was conducted to examine the role of baseline PMP in response to training and quality control. Based on initial PMP, OPUs were categorized into low‐performance (< 5 PMP), medium‐performance (5–10 PMP), and high‐performance (> 10 PMP) groups. The results showed that the low‐performing units experienced the most substantial short‐term improvement after training (from 1.55 to 7.91 within 3 months), but this dropped to 5.06 within 6 months unless reinforced with IrOQS. The high‐performing units maintained their performance longer but also experienced a decline in the absence of continued oversight. After IrOQS, all groups showed renewed improvement, especially the low‐performing group, whose PMP rose to 8.34. These findings suggest that although training is effective at all levels, the sustainability of its impact is highly dependent on continuous reinforcement. Implementing targeted interventions and periodic training programs for weaker units is essential to maintain previous gains.

The results of the change point detection analysis provided valuable insights into the temporal dynamics of performance improvement after training interventions. The results of this analysis showed that most OPUs showed at least one detectable change in their PMP trajectory after IrOSS. Notably, high‐ and medium‐performing units showed almost complete response rates to IrOSS and IrOQS, suggesting that these units may also respond positively to enhanced training programs. In contrast, the low‐performing units showed a good initial response to IrOSS but a less promising response to IrOQS.

It is worth noting that our study was conducted across 25 OPUs distributed throughout Iran, reflecting real‐world implementation conditions. However, this modest sample size, combined with substantial interunit heterogeneity—particularly in units such as Yasouj and Rafsanjan—limits the statistical power of our analyses and constrains the generalizability of findings to broader national contexts. This heterogeneity likely stems from regional disparities in healthcare infrastructure, cultural attitudes toward organ donation, and variations in local implementation fidelity. Future multicenter studies with larger, more homogeneous samples would be required to validate these findings and identify contextual factors that moderate intervention effectiveness.

Also, an important observation from our study is that none of the 25 OPUs met the a priori criteria for sustained improvement, despite exposure to both initial training (IrOSS) and quality reinforcement sessions (IrOQS). This pattern suggests that episodic training alone may be insufficient to generate permanent shifts in performance. Rather than indicating intervention failure, this finding reveals an important systems‐level insight: Organ donation performance appears to function as a reversible state that requires continuous reinforcement to maintain. Without continuous support, performance naturally regresses toward baseline once external stimuli are removed. This observation carries critical implications for policy: Sustainability may require transforming IrOQS from an episodic event into a continuous quality infrastructure embedded within routine OPU operations. Future implementation strategies should prioritize system integration over repeated training to achieve lasting change.

Findings from all analyses reveal a common challenge. Although training interventions are effective in the short term, the lack of sustained improvements across all clusters suggests that retraining sessions are essential to consolidate and maintain performance.

## 5. Conclusion

Given the critical role of OPUs in managing the organ donation process and the importance of training and evaluation in optimizing their performance, this study aimed to assess the effectiveness of the IrOSS and IrOQS programs on OPU performance.

The main strength of this dual approach is that it combines education with ongoing quality control, directly addressing the limitations raised in previous studies on the sustainability of educational effects. The two educational systems implemented, IrOSS and IrOQS, seek to maintain and improve organ donation rates over time by establishing a framework that includes periodic assessments and targeted retraining. Previous research has shown that without such periodic measures, improvements in organ donation performance are often lost, as was observed in our study in the control group that did not receive periodic follow‐up intervention.

This research also faced limitations in its implementation that may affect the interpretation of its findings. These include the following:

− The number of study units was limited, and the results may not be generalizable to all centers.
− Unexamined or uncontrolled variables in this study, such as regional and cultural conditions, may have influenced the results obtained.

Given these limitations, future research should aim for more comprehensive assessments and consider more variables that influence the training process. In addition, it is important to identify the optimal timing for retraining to maximize sustained improvements.

## Funding

No funds, grants, or other support was received.

## Conflicts of Interest

The authors declare no conflicts of interest.

## Data Availability

The datasets supporting the conclusions of this article will be made available by the authors.

## References

[1] Sakallı G. D. and Dağ G. S. , Organ Transplantation and Donation from the Point of View of College Students, Paper presented at the Transplant Proc. (2020) . 10.1016/j.transproceed.2019.11.008 31901318

[2] Lewis A. , Koukoura A. , Tsianos G. I. , Gargavanis A. A. , Nielsen A. A. , and Vassiliadis E. , Organ Donation in the US and Europe: the Supply vs Demand Imbalance, Transplantation Reviews. (2021) 35, no. 2, 10.1016/j.trre.2020.100585. 33071161

[3] Iranian Society of Organ Donation 2021, 2022, https://ehdacenter.ir/archive/article/2759553.

[4] IRODaT-International Registry on Organ Donation and Transplantation, 2022, https://www.irodat.org/?p=database.

[5] Chuang Y.-M. , Yeh S.-S. , Tseng C.-F. , and Tseng C.-C. , Soliciting Organ Donations by Medical Personnel and Organ Donation Coordinators: a Factor Analysis, PLoS One. (2021) 16, no. 4, 10.1371/journal.pone.0250249. PMC8064528 33891641

[6] Santiago C. , Gómez P. , Olivares J. , and de La Concepción M. , Evaluation of Organ Procurement in an Area Under the Influence of a Training Program, Transplantation Proceedings. (2005) 37, no. 9, 3649–3650, 10.1016/j.transproceed.2005.08.056, 2-s2.0-29544435526. 16386493

[7] Czerwiński J. , Jakubowska-Winecka A. , Woderska A. et al., Implementation and Sustainability of European Training Program on Organ Donation in Poland: Results and the Impact on Donation Indicators, Transplantation Proceedings. (2016) 48, no. 7, 2429–2433, 10.1016/j.transproceed.2015.12.144, 2-s2.0-84992435586. 27742315

[8] Potter J. E. , Elliott R. M. , Kelly M. A. , and Perry L. , Education and Training Methods for Healthcare Professionals to Lead Conversations Concerning Deceased Organ Donation: an Integrative Review, Patient Education and Counseling. (2021) 104, no. 11, 2650–2660, 10.1016/j.pec.2021.03.019. 33775500

[9] Heo S. J. , Ju Y. H. , Noh E. J. et al., A Study on the Performance of the Donation Improvement Program in Korea, Korean Journal of Transplantation. (2021) 35, no. 2, 77–85, 10.4285/kjt.21.0006. 35769527 PMC9235343

[10] Siminoff L. A. , Marshall H. M. , Dumenci L. , Bowen G. , Swaminathan A. , and Gordon N. , Communicating Effectively About Donation: An Educational Intervention to Increase Consent to Donation, Progress in Transplantation. (2009) 19, no. 1, 35–43, 10.1177/152692480901900105. 19341061

[11] Shu W. , Xing B.-y. , Ruan W.-x. , Gao L.-y. , and Miao Q.-f. , Exploring the Relationship Between Professional Identity and Psychological Resilience of Organ Donation Coordinators in Zhejiang Province (China), Frontiers in Public Health. (2021) 746. 10.3389/fpubh.2021.659871 PMC8290186 34295865

[12] Manyalich M. , Guasch X. , Paez G. , Valero R. , and Istrate M. , ETPOD (European Training Program on Organ Donation): A Successful Training Program to Improve Organ Donation, Transplant International. (2013) 26, no. 4, 373–384, 10.1111/tri.12047, 2-s2.0-84875382308. 23279320

[13] de Andrade J. and Figueiredo K. F. , Impact of Educational and Organizational Initiatives in Organ Donation in a Southern Brazilian State in the Last Decade, Transplantation Proceedings. (2019) 51, no. 3, 625–631, 10.1016/j.transproceed.2018.10.033, 2-s2.0-85063972303. 30979444

[14] Paez G. , Valero R. , and Manyalich M. , Training of Health Care Students and Professionals: a Pivotal Element in the Process of Optimal Organ Donation Awareness and Professionalization, Transplantation Proceedings. (2009) 41, no. 6, 2025–2029, 10.1016/j.transproceed.2009.05.020, 2-s2.0-68949204229. 19715824

